# Is inflammation a new risk factor of depression in haemodialysis patients?

**DOI:** 10.1007/s11255-012-0269-y

**Published:** 2012-09-13

**Authors:** Łukasz Nowak, Marcin Adamczak, Andrzej Więcek

**Affiliations:** Department of Nephrology, Endocrinology and Metabolic Disorders, Medical University of Silesia, Francuska 20-24, 40-027 Katowice, Poland

**Keywords:** Chronic kidney disease, Haemodialysis, Depression, Inflammation

## Abstract

**Purpose:**

Prevalence of depression is high in patients with chronic kidney disease. Depression is associated with increased mortality and the higher rate of suicides in these patients. The aim of the study was to estimate the prevalence of depressive symptoms in haemodialyzed patients in Upper Silesia region of Poland and the possible role of inflammation in depression development.

**Methods:**

Six hundred and ninety-seven haemodialyzed patients from 22 dialysis centres in Upper Silesia region of Poland were enrolled into the study. Mean age was 59.1 ± 0.5 years, and mean time of dialysis treatment was 3.6 ± 0.2 years. Each patient received 21-item Beck Depression Inventory (BDI) questionnaire for depression screening. Additional questions considering length of dialysis treatment, concomitant diseases and number of days spent in hospitals during the last year were also asked.

**Results:**

Depressive symptoms were found in 268 (38.6 %) patients. Patients with depressive symptoms when compared with the patients without them tended to have higher C-reactive protein plasma concentration (14.3 ± 1.3 vs. 11.1 ± 0.9 mg/l; *p* = 0.067) and were more often dialyzed with central catheter (27.6 vs. 18.2 %; *p* = 0.0042). During the last year, patients with depressive symptoms spent in hospitals more days than patients without depressive symptoms (24.3 vs. 15.3 days, respectively; *p* < 0.0001). Significant positive correlation between BDI score and C-reactive protein level (*r* = 0.1625; *p* = 0.001) was found both in univariate and multivariate analysis.

**Conclusions:**

(1) Depressive symptoms are frequently found (38.6 %) in haemodialyzed patients in Upper Silesia part of Poland. (2) Catheter placement and inflammation seem to play an important role in the pathogenesis of depression in haemodialysis patients.

## Introduction

Depression issue in haemodialyzed patients has been addressed many times in the past, and by now, we may expect about 3,000 haemodialyzed patients diagnosed for depression described in the literature through the last 30 years. During this time, a rapid and pronounce development in renal replacement therapy was observed which was followed by deep change in profile of haemodialyzed population. However, depression rate in chronic kidney disease (CKD) patients does not differ from general population [1], and reported prevalence of depression in ESRD patients is much higher than in general population [2], did not change over time [3–9] and is the most common psychiatric disorder in this group of patients [10]. The diagnosis of depression still remains challenging which was shown by Donna Grant in 2008 on British population of haemodialyzed patients [11].

Depression negatively influences compliance with medical treatment and dietary adherence in different groups of chronically ill patients, including patients with CKD and ESRD [12–14]. That in turn can be associated with poor outcomes in CKD patients independently of other clinical factors like CKD stage, serum albumin, haemoglobin blood or phosphorus plasma concentrations and comorbidities [15, 16]. It was proven that depression influences the lifetime of patients with CKD [17–19] and may affect suicide rate in this population [20].

Etiopathology of depression in patients with CKD is multifactorial, both medically and psychologically based. Theory of *major loses* explains both backgrounds for depression development. Sense of loss accompanies patients from very early stages of CKD to the most profound loss in ESRD. Additional factors also influence development of depression in ESRD patients. Chronic pain and sleep disturbances affect health-related quality of life (HRQOL) and depression in patients with all stages of CKD [21] and with ESRD [22–24], with regard to not clear causality and order. Additionally, numerous authors underline role of patient’s inflammatory status in depression development [25–28].

Since there is little data about prevalence of depression in haemodialyzed population in Poland, we designed a study on large group of haemodialyzed patients to address this issue. We also wanted to identify potential risk factors for depression development in this population with regard to inflammatory status. The question arises: is depression a new player in MIA syndrome? Confirming this hypothesis may result in changing MIA to MIAD syndrome abbreviation (malnutrition–inflammation–atherosclerosis–depression) in the future.

## Materials and methods

Six hundred and ninety-four haemodialyzed patients from 22 dialysis centres in Upper Silesia region of Poland were enrolled into the study. Patients’ characteristics were shown in Tables 1 and 2. Each patient received questionnaire consisting of the parts: Beck Depression Inventory (BDI) test, social data and medical history data.

**Table 1:** Characteristics—clinical data of dialyzed patients with chronic kidney disease

Number of patients	694
Gender	
Male	387 (55.7 %)
Female	307 (44.3 %)
Age (years)	59.1 ± 0.5
Weight (kg)	71.2 ± 0.6
Height (cm)	166.5 ± 0.4
Haemodialysis time per week (h)	12.5 ± 0.2
Haemodialysis time (years)	3.6 ± 0.2
Vascular access	
Venous catheter	159 (24.2 %)
Arterio-venous fistula	535 (75.8 %)
Dialysis centre	
Non-profit	434 (63.4 %)
Commercial	260 (36.6 %)
Cause of CKD^a^	
Diabetes mellitus	148 (21.2 %)
Hypertension	284 (40.7 %)
Glomerulonephritis	122 (17.5 %)
Other	96 (13.8 %)
Other diseases^a^	
Chronic pulmonary obstructive disease	17 (2.4 %)
Coronary artery disease	166 (23.8 %)
History of myocardial infarction	83 (11.9 %)
History of stroke	43 (6.2 %)
Glaucoma	45 (6.5 %)
Hearing problems	109 (15.6 %)
Laboratory results	
Haemoglobin (g/dl)	11.0 ± 0.1
Phosphate (mmol/l)	1.8 ± 0.1
Protein (g/l)	67.7 ± 0.3
Cholesterol (mmol/l)	4.7 ± 0.1
C-reactive protein (mg/l)	11.7 ± 0.7

Mean values ± SEM

^a^Self reported by patients

**Table 2:** Characteristics—social and other data of dialyzed patients with chronic kidney disease

Marriage status	
Married	434 (62.3 %)
Alone	126 (18.1 %)
Widowed	127 (18.2 %)
Living conditions	
With family	585 (83.9 %)
Alone	98 (14.1 %)
Education	
Elementary	270 (38.7 %)
High school	349 (50.1 %)
College	66 (9.5 %)
Number of pills taken daily	10.7 ± 0.2
Number of days spent in hospital during the last year	18.7 ± 0.9
BDI score	14.7 ± 0.4

Mean values ± SEM

*BDI* beck depression inventory

Presence of depressive symptoms was measured with BDI [29] that is a well-validated measure of depression in patients with ESRD [4]. The BDI is a 21-item, patient self-rated scale that has been used in haemodialyzed patients and correlates highly with diagnostic criteria for depression, quality of life, functional status, severity of illness and mortality over time [30, 31]. The BDI test uses 0–3 Likert scales, with total scores ranging from 0 to 63. Higher scores correlate with more severe depression. BDI score >16 is characteristic for presence of moderate to severe depressive symptoms.

Social part of questionnaire considered marriage status, education and living conditions.

Medical history considered: primary illness as cause of CKD, additional illnesses, length of dialysis treatment, ownership type of dialysis centre (non-profit or commercial), type of vascular access, number of days spent in hospitals during the last year and number of pills taken daily.

In each patient, the following laboratory results were obtained: blood haemoglobin concentration, serum protein, cholesterol and C-reactive protein concentrations. For these data collections, questionnaires were temporarily unblinded locally in dialysis centres.

Age, weight, height and gender of each patient were also obtained.

Participation in this study was voluntary and anonymous although good understanding of given questions was obligatory. Only complete questionnaires were investigated.

Statistical analysis was made with Statistica 5.0 PL program. Data were presented as means and standard error of means (SEM). Differences between groups were calculated with *T* Student and χ^2^ tests. Correlation analysis was performed with Pearson’s test and multiple regression test.

## Results

Overall BDI score for studied population was 14.7 ± 0.4 points. Moderate to severe depressive symptoms of depression (BDI > 16) were found in 268 (38.6 %) patients of studied population. Detailed comparison between groups with or without depression is shown in Table 3a, b.

**Table 3:** Characteristics and comparison between groups of haemodialyzed patients with ESRD with or without depressive symptoms

	Depressive symptoms group	Non-depressive symptoms group	*p*
*a*			
Number of patients	268 (38.6 %)	426 (61.4 %)	
BDI score	24.8	5.4	
Age (years)	61.8 ± 0.8	57.5 ± 0.7	**0.002**
Weight (kg)	71.7 ± 0.9	70.8 ± 0.7	0.438
Gender			
Male	141 (52.6 %)	246 (57.6 %)	0.185*
Female	127 (47.4 %)	180 (42.4 %)	
Haemodialysis time per week (h)	12.8 ± 0.4	12.6 ± 0.2	0.227
Haemodialysis time (years)	3.5 ± 0.3	3.7 ± 0.2	0.616
Vascular access			
Venous catheter	77 (27.6 %)	82 (18.2 %)	0.004*
Arterio-venous fistula	191 (72.4 %)	344 (80.8 %)	
Dialysis centre			
Non-profit	186 (70.2 %)	248 (58.8 %)	0.003*
Commercial	82 (29.8 %)	178 (41.2 %)	
Cause of CKD^a^			
Diabetes mellitus	67 (24.6 %)	81 (19.1 %)	0.060*
Hypertension	121 (44.5 %)	163 (38.4 %)	0.072*
Glomerulonephritis	40 (14.7 %)	82 (19.3 %)	0.145*
Other	39 (14.3 %)	57 (13.4 %)	
Other diseases^a^			
Chronic pulmonary obstructive disease	11 (4.0 %)	6 (1.4 %)	0.025*
Coronary artery disease	85 (31.3 %)	81 (19.1 %)	0.001*
History of myocardial infarction	42 (15.4 %)	41 (9.6 %)	0.017*
History of stroke	28 (10.3 %)	15 (3.5 %)	<0.001*
Glaucoma	22 (8.1 %)	23 (5.4 %)	0.143*
Hearing problems	50 (18.4 %)	59 (13.9 %)	0.090*
Laboratory results			
Haemoglobin (g/dl)	10.9 ± 0.1	11.2 ± 0.2	0.166
Phosphate (mmol/l)	1.8 ± 0.0	1.8 ± 0.0	0.845
Protein (g/l)	66.8 ± 0.6	68.2 ± 0.4	0.058
Cholesterol (mmol/l)	4.6 ± 0.1	4.7 ± 0.1	0.286
C-reactive protein (mg/l)	14.3 ± 1.3	11.1 ± 0.9	0.067
*b*			
Marriage status			
Married	157 (59.5 %)	277 (65.5 %)	**0.004**
Alone	42 (15.9 %)	84 (19.9 %)	
Widowed	65 (24.6 %)	62 (14.7 %)	
Living conditions			
With family	219 (83.3 %)	366 (87.1 %)	0.160*
Alone	44 (16.7 %)	54 (12.9 %)	
Education			
Elementary	117 (44.0 %)	153 (36.5 %)	**0.027**
High school	132 (49.6 %)	217 (51.8 %)	
College	17 (6.4 %)	49 (11.7 %)	
Number of pills taken daily	10.9 ± 0.3	10.6 ± 0.3	0.490
Number of days spent in hospital during the last year	24.3 ± 1.8	15.3 ± 1.0	**<0.001**

Mean values ± SEM

* χ^2^ test

^a^Self reported by patients

Bold values indicate statistical differences between sub-groups

If compared with patients without depressive symptoms, patients with depressive symptoms were older (61.7 ± 14.2 vs. 57.5 ± 14.4 years, respectively; *p* < 0.001), spent more days in hospitals during the last year (24.3 vs. 15.3 days, respectively; *p* < 0.001) and tended to have higher C-reactive protein plasma concentration (14.3 ± 1.3 vs. 11.1 ± 0.9 mg/l, respectively; *p* = 0.067). Amongst patients with depressive symptoms there were more dialyzed ones with central catheter than in group without depressive symptoms (28 vs. 19 %, respectively; *p* = 0.004) and more patients were treated by non-profit dialysis centres (70 vs. 59 %, respectively; *p* = 0,003). Patients dialyzed in non-profit dialysis centres spent more days in hospital during the last year than patients dialyzed in commercial ones (21.6 vs. 13.8 days, respectively; *p* < 0.001).

In group with depressive symptoms there were more widowed patients than in group without depressive symptoms (24 vs. 13 %, respectively). Considering all investigated population, widowed patients had higher BDI score than married and lonely patients (17.7 vs. 14.3 points, respectively; *p* < 0.001 and vs. 13.0 points; *p* < 0.001, respectively). Married and lonely patients were not different with regard to BDI score (14.3 vs. 13.0 points, respectively; *p* = 0.162).

There were also differences in education level—in the group with depressive symptoms there were 44/50/6 % of patients after elementary, high schools and college comparing to 37/52/11 % in the group without depressive symptoms, respectively (*p* = 0.027). Considering all investigated population, patients after college had significantly lower BDI score than patients after elementary school—12.7 vs. 15.7 points (*p* = 0.029).

There were no significant differences between groups with regard to gender and family status (living alone or with family).

If compared with patients with arterio-venous fistula, the group of patients dialyzed with central venous catheter were characterized by significantly higher C-reactive protein serum concentration (15.9 vs. 11.0 mg/l; *p* = 0.013) and BDI score (16.7 vs. 14.0 points; *p* = 0.002) (Table 4). This group was characterized also by more days spent in hospital during the last year (28.9 vs. 15.6; *p* < 0,001) and shorter dialysis treatment time (2.9 vs. 3.8 years; *p* = 0.025).

**Table 4:** Comparison between patients dialyzed with the usage of arterio-venous fistula vs. central venous catheter

	Arterio-venous fistula group	Central venous catheter group	*p*
Number of patients	528 (76.1 %)	166 (23.9 %)	
Depression (BDI ≥16)	189 (35.8 %)	75 (45.2 %)	0.029*
BDI score	14.0 ± 0.4	16.7 ± 0.8	**0.002**
Age (years)	57.9 ± 0.6	63.0 ± 1.1	**<0.001**
Haemodialysis time (years)	3.8 ± 0.2	2.9 ± 0.4	**0.025**
Laboratory results			
Haemoglobin (g/dl)	11.1 ± 0.2	11.0 ± 0.1	0.682
C-reactive protein (mg/l)	11.0 ± 0.7	15.9 ± 2.0	**0.013**
Number of days spent in hospital during the last year	15.6 ± 0.9	28.9 ± 2.4	**<0.001**

Analysis of whole investigated population (*n* = 694). Mean values ± SEM

* χ^2^ test

Bold values indicate statistical differences between sub-groups

Significant positive correlations between BDI score and age (*r* = 0.152; *p* = 0.002), days spent in hospitals during the last year (*r* = 0.210; *p* < 0.001) and C-reactive protein level (*r* = 0.162; *p* = 0.001) were found in entire studied population. Also multivariate regression analysis was performed with BDI score as dependent variable and independent variables: gender, age, time spent on dialysis treatment, type of vascular access, type of dialysis unit, marriage status, living conditions, educational level, number of pills taken daily, days spent in hospitals during the last year, blood concentrations of haemoglobin, plasma concentration of phosphate, cholesterol, protein and CRP. The multivariate regression analysis showed that BDI score depends on age (*p* = 0.019; beta = 0.117), days spent in hospitals during the last year (*p* = 0.004; beta = 0.141) and C-reactive plasma concentration (*p* = 0.037; beta = 0.099).

## Discussion

Based on the results obtained in this study, which include the largest population of haemodialyzed patients diagnosed for the presence of depression in Poland, we can conclude that (a) depressive symptoms are frequent (38 %) and their presence mainly depends on time spent in hospitals and (b) presence of central catheter can be used as a simple marker to identify patients with higher risk for depression development.

Depressive symptoms rate in studied population is not different than reported before in many other studies [3, 13, 15, 30, 31]. We have anticipated that family-based culture as Polish may have some influence on depression presence in severely ill patients. It seems that cultural differences between dialyzed patients’ populations (European, American, Afro-American, Taiwan, Japanese) do not affect depression development, which proves how profound impact on patients HRQOL, CKD stage 5.

Correlation between presence of depressive symptoms and time spent in hospitals during the last year may be bidirectional. It was already found that depression in haemodialyzed patients is associated with the higher rate of hospital admissions [32]. There are no data if treatment of depression can decrease hospitalization rate. And we do not know whether shortening time spent on treatment in hospital will decrease depression rate in haemodialyzed population. We assume that time spent in hospitals reflects overall patient’s health status, seriousness of comorbidities and need for longer treatment. Causality in these circumstances is very hard to establish.

The difference in percentage of patients with depressive symptoms between profit and non-profit dialysis units requires wider comment. As we understand, there are two explanations of this finding. First is that the source of the patients of small non-profit dialysis units (which are in majority right now) is not large nephrological, emergency and cardiology public wards. Such sources are mainly outpatients’ clinics. Second is that their deal with national health provider does not sufficiently cover expenses on seriously ill patients who stay longer in hospitals. These two reasons may create the situation, when in non-profit dialysis units there are more ill patients with lots of comorbidities and obviously higher rate of depression than in public centres. So, the difference in depressive symptoms rate between profit and non-profit dialysis units probably results from patients’ allocation and reflects status of our healthcare system in this area.

Thus, it is established that during the first year after start of dialysis treatment, there is a higher rate of depression and suicides in this population [30]. This time is also characterized by wider usage of central catheters. Other subpopulation of haemodialyzed patients are those with problems with vascular access, with history of many catheters used and with many vascular changes. Our study did not make a difference between these two subgroups. All catheter bearers were identified as a group with significantly higher BDI score, higher C-reactive protein plasma concentration and more days spent in hospital during the last year. Such a correlation was not observed in 109 patients investigated by Leinau et al. [33]. Although, there is connection between presence of a central catheter and higher possibility of infection and inflammation, we conclude that higher plasma C-reactive protein level in patients with central catheter reflects overall worse inflammatory status of these patients which implicate more intensive treatment in hospitals.

First episode of depression in general population mainly occurs below 30 years of age [34] and its prevalence declines with the age. In our population, depression was positively correlated with age, and time of dialysis treatment was not different in groups with or without depressive symptoms. This is contrary to previous findings in which depression is mainly bound to the first 12 months of dialysis treatment with the higher rate of suicides, especially in younger patients. This difference could be explained by “advanced” average age of whole investigated population and their marital status, which can be characterized as “middle-aged”, in majority married (62 %) and living with family (84 %). That obviously delivers enough social support. On the other hand, it is understandable that the longer patients are dialyzed, the higher rate of complications and comorbidities occurs, with higher possibility of depression development.

One of the more surprising findings was the same rate of depression in haemodialyzed man and woman. It is known that depression is much more frequent (2 times) in woman than in man all over the world in different cultures [35]. This is related mainly to higher stress rate and lower amount of inner resources in woman. It seems that overall burden connected to chronic kidney disease and dialysis treatment levels the stress rate in men and woman, and gender differences stop to play an important role in depression development. This could be rather due to rise of stress level in man than decrease stress in woman in dialysis population. This is the only speculation, because this area was not investigated.

Recently, many authors have tried to find a connection between depression and aggravated inflammatory status of patients with ESRD [36]. It was proven that proinflammatory cytokines play an important role in the pathogenesis of depression in general population [37, 38]. The same cytokines are elevated in ESRD patients [39, 40] and may predict mortality in this group [41]. Also C-reactive protein (CRP) concentration was used to predict outcome in haemodialysis patients [42]. Amongst others, interleukin-6 (IL-6) became most often used marker to link depression and inflammation in patients with CKD, ESRD and after kidney transplantation [40, 43, 44]. Elevated IL-6 level may increase risk of depression development in patients with ESRD [45, 46]. Causality of depression and inflammation is unclear, probably bilateral. It was found that depression is linked to worse nutritional status of patients with ESRD [25] that is the part of malnutrition–inflammation–atherosclerosis (MIA) syndrome. Salawa and Omima showed in 60 haemodialysis patients the link between depressive symptoms, poor quality of life and malnutrition–inflammation complex assessed with MIA [42]. Additionally, depression is associated with higher cardiovascular risk in patients with ESRD [26–28], which also could be mediated through depression-aggravated atherosclerosis.

Our study has several limitations. We did not include all patients dialyzed in our region (30 % of all of them were included). Those who could not or did not want to participate were not investigated. This certainly underestimated the presence of depression as non-compliance is a depression marker, and as inability to fulfil questionnaire reflects much worse general state of patient. We did not collected data about work activities and being on waiting list for transplantation. We may assume that working patients and those who await transplantation have better (lower) BDI score. We chose C-reactive protein as a marker for inflammation because of its low cost in regard to large studied population, although as presented recently in many papers, IL-6 seems to be a better marker. The data concerning cause of CKD and comorbidities were obtained from the patients—so credibility of this data may be questioned. We did not investigated presence of pain, fatigue and quality of sleep in studied population. They were proven in other studies to affect depression rate in haemodialyzed population.

In summary, this study once again proved high prevalence of depressive symptoms in haemodialyzed patients. We confirmed a link between depression and inflammatory status in large group of patients, and we identified a subpopulation of catheter bearers as a group with high risk for depression development. The efforts should be made to shorten inpatients’ treatment time to decrease occurrence of severe depression. One of the ways to achieve this is early diagnosis of CKD and starting dialysis treatment with already ready-to-puncture arterio-venous fistula. We suggest considering usage of MIAD syndrome instead of MIA abbreviation but this idea requires further studies.

Conclusions: 1. Depressive symptoms are frequently found (38.6 %) in haemodialyzed patients in Upper Silesia part of Poland. 2. Catheter placement and inflammation seem to play an important role in the pathogenesis of depression in haemodialysis patients.
